# Supplementary figures and images for: Public perceptions of synthetic cooling agents in electronic cigarettes on Twitter
Source: PLoS One. 2024 Mar 12;19(3):e0292412. doi: 10.1371/journal.pone.0292412 (PMC10931480; doi:10.1371/journal.pone.0292412)

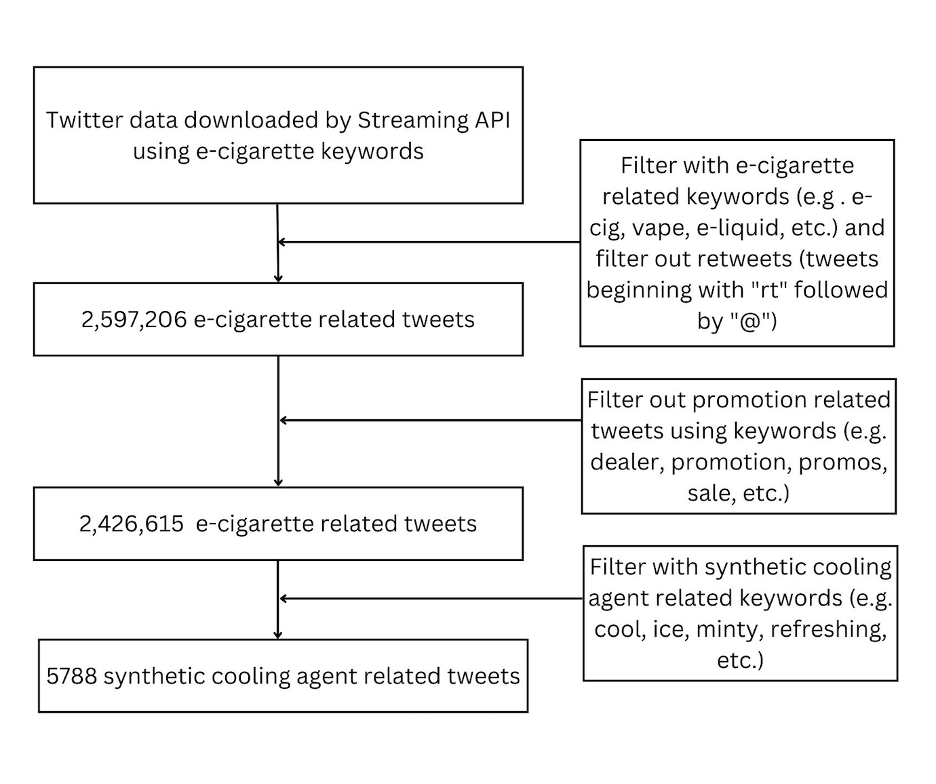

Supplement: S1 Fig — (TIF) [file pone.0292412.s001.tif]

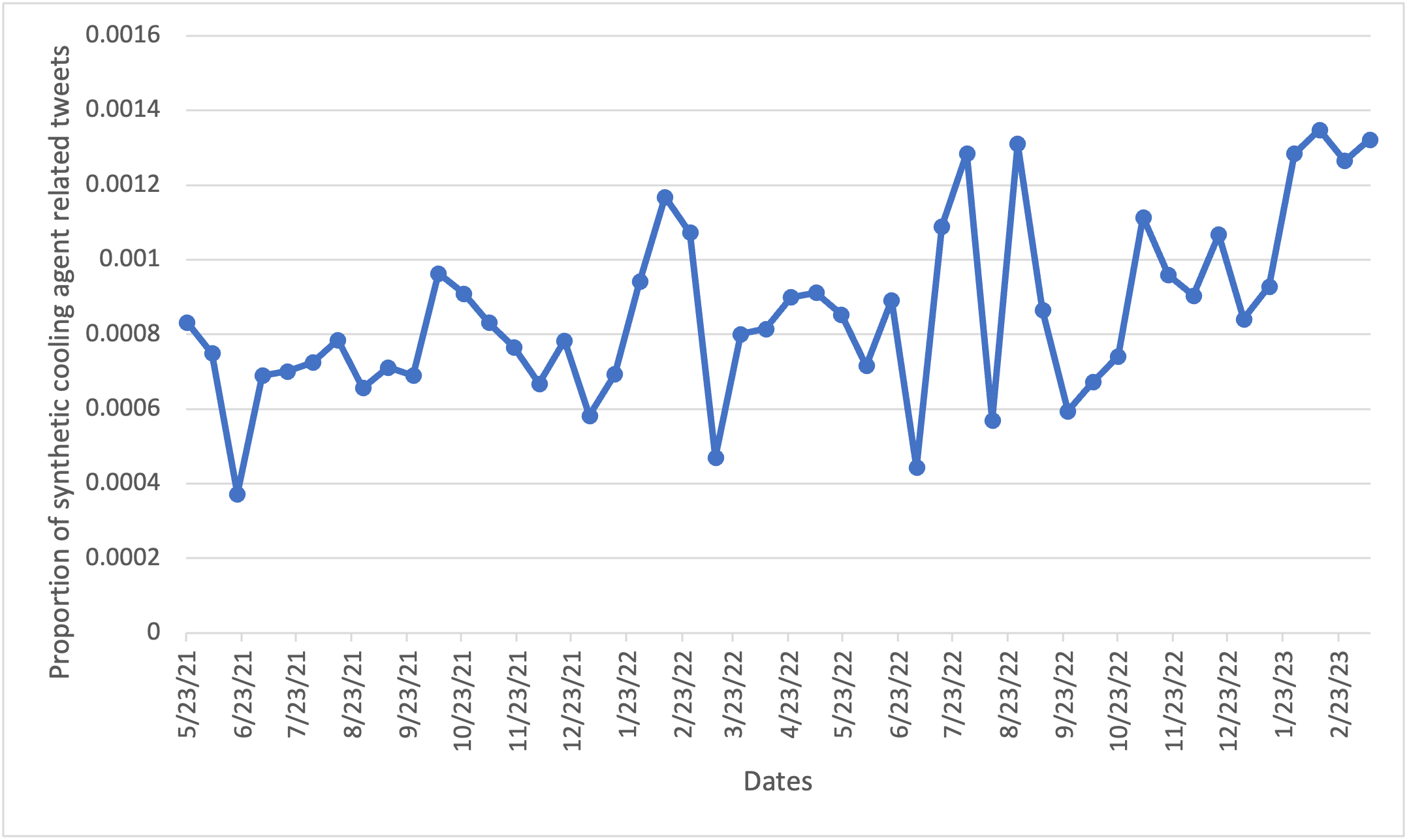

Supplement: S2 Fig — (TIF) [file pone.0292412.s002.tif]

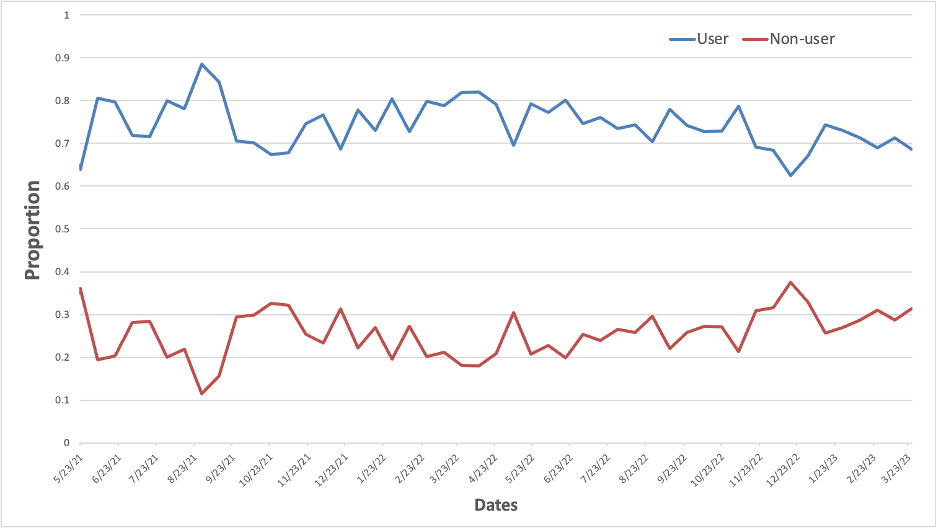

Supplement: S3 Fig — (TIF) [file pone.0292412.s003.tif]
