# Supplementary material for: Public perceptions of synthetic cooling agents in electronic cigarettes on Twitter
Source: PLoS One. 2024 Mar 12;19(3):e0292412. doi: 10.1371/journal.pone.0292412 (PMC10931480; doi:10.1371/journal.pone.0292412)
Supplement: S1 Table — (DOCX) [file pone.0292412.s004.docx]

**S1 Table. Keywords used in Twitter data filtering.**

| **Positive Keywords** | **Negative Keywords** |
| --- | --- |
| cooling | Ice cream |
| coolness | Coffee |
| ice | Iced coffee |
| icy | Iced Coffees |
| iced | Ice Tea |
| minty | Iced Tea |
| koolada | Dry Ice |
| WS-23 | Ice Latte |
| WS-5 | Iced lattes |
| WS-3 | Iced chai |
| frosty | Ice americano |
| refreshing | Iced americano |
| frost | Ice Water |
| refreshed | Iced Water |
| nippy | Iced Caramel |
| chilled | Smirnoff Ice |
| snowy | Frozen Yogurt |
| arctic | Kool kids |
| glacial | Kool kid |
| freshness | Cool kids |
| coolness | Ice Breaker |
| popsicle | Ice Cold Water |
|  | Ice Cube |
|  | Ice Cubes |
|  | Frozen Water |
|  | Snowy Mountains |
|  | Dunkin Iced |
|  | Arctic Monkeys |
|  | Kool aid |
|  | Ice Spice |
|  | Natty Ice |
|  | Chilled out |
|  | Cooling tunnel |
|  | Ice Cold La Croix |
|  | Ice Packs |
|  | Ice Cold Slushie |
|  | Crushed Ice |
|  | Ice Bucket |
|  | Ice Cold Diet Coke |
|  | #MHHSBD |
|  | #vapenation |
|  | Ice box |
